# Cholecystectomy is associated with higher risk of recurrence after microwave ablation of hepatocellular carcinoma: a propensity score matching analysis

**DOI:** 10.20892/j.issn.2095-3941.2019.0246

**Published:** 2020-05-15

**Authors:** Hongcai Yang, Yi Yang, Jianping Dou, Rui Cui, Zhigang Cheng, Zhiyu Han, Fangyi Liu, Xiaoling Yu, Xiang Zhou, Jie Yu, Ping Liang

**Affiliations:** ^1^School of Medicine, Nankai University, Tianjin 300071, China; ^2^Department of Interventional Ultrasound, Chinese PLA General Hospital, Beijing 100853, China; ^3^Department of Interventional Therapy, National Cancer Center/National Clinical Research Center for Cancer/Cancer Hospital, Chinese Academy of Medical Sciences and Peking Union Medical College, Beijing 100021, China

**Keywords:** Cholecystectomy, microwave ablation, hepatocellular carcinoma, propensity score matching

## Abstract

**Objective:** To explore the association between cholecystectomy and the prognostic outcomes of patients with hepatocellular carcinoma (HCC) who underwent microwave ablation (MWA).

**Methods:** Patients with HCC (*n* = 921) who underwent MWA were included and divided into cholecystectomy (*n* = 114) and non-cholecystectomy groups (*n* = 807). After propensity score matching (PSM) at a 1:2 ratio, overall survival (OS) and disease-free survival (DFS) rates were analyzed to compare prognostic outcomes between the cholecystectomy (*n* = 114) and non-cholecystectomy groups (*n* = 228). Univariate and multivariate Cox analyses were performed to assess potential risk factors for OS and DFS. Major complications were also compared between the groups.

**Results:** After matching, no significant differences between groups were observed in baseline characteristics. The 1-, 3-, and 5-year OS rates were 96.5%, 82.1%, and 67.1% in the cholecystectomy group, and 97.4%, 85.2%, and 74.4% in the non-cholecystectomy group (*P* = 0.396); the 1-, 3-, and 5-year DFS rates were 58.4%, 34.5%, and 26.6% in the cholecystectomy group, and 73.6%, 44.7%, and 32.2% in the non-cholecystectomy group (*P* = 0.026), respectively. The intrahepatic distant recurrence rate in the cholecystectomy group was significantly higher than that in the non-cholecystectomy group (*P* = 0.026), and the local tumor recurrence and extrahepatic recurrence rates did not significantly differ between the groups (*P* = 0.609 and *P* = 0.879). Multivariate analysis revealed that cholecystectomy (HR = 1.364, 95% CI 1.023–1.819, *P* = 0.035), number of tumors (2 *vs.* 1: HR = 2.744, 95% CI 1.925–3.912, *P* < 0.001; 3 *vs.* 1: HR = 3.411, 95% CI 2.021–5.759, *P* < 0.001), and γ-GT levels (HR = 1.003, 95% CI 1.000–1.006, *P* < 0.024) were independent risk factors for DFS. The best γ-GT level cut-off value for predicting median DFS was 39.6 U/L (area under the curve = 0.600, *P* < 0.05). A positive correlation was observed between cholecystectomy and γ-GT level (*r* = 0.108, 95% CI −0.001–0.214, *P* = 0.047). Subgroup analysis showed that the DFS rates were significantly higher in the non-cholecystectomy group than the cholecystectomy group when γ-GT ≥39.6 U/L (*P* = 0.044). The 5-, 10-, 15-, 20-, and 25-year recurrence rates from the time of cholecystectomy were 2.63%, 21.93%, 42.11%, 58.77%, and 65.79%, respectively. A significant positive correlation was observed between cholecystectomy and the time from cholecystectomy to recurrence (*r* = 0.205, 95% CI 0.016–0.379, *P* = 0.029). There were no significant differences in complications between groups (*P* = 0.685).

**Conclusions:** Patients with HCC who underwent cholecystectomy were more likely to develop intrahepatic distant recurrence after MWA, an outcome probably associated with increased γ-GT levels. Moreover, the recurrence rates increased with time.

## Introduction

Hepatocellular carcinoma (HCC) is one of the most common malignancies in China, with 364,800 new cases and 318,800 death cases in 2015, which accounted for more than 50% of the newly diagnosed cases and deaths worldwide^1,2^. Surgical resection and orthotopic liver transplantation, which are accepted as radical therapies for HCC, are limited by cirrhosis, poor hepatic reserves, advanced tumor progression, and the scarcity of donated organs^3^. Minimally invasive thermal ablation of HCC has become common since the advent of modern imaging and is recommended by the American Association for the Study of Liver Diseases (AASLD), European Association for the Study of the Liver (EASL), Asian Pacific Association for the Study of the Liver (APASL), and National Comprehensive Cancer Network (NCCN) guidelines as an effective therapy for HCC^4–7^. Microwave ablation (MWA) has been established as a frequently used and acceptable therapeutic method for HCC treatment, according to the Milan criteria, and has been associated with many theoretical advantages, such as higher intra-tumoral temperature, greater ablation volume, and shorter operation time than those of radiofrequency ablation (RFA).

Cholecystectomy has been used as a therapeutic surgery to treat gallbladder-related diseases for more than a century, and it is thought to be associated with the declining morbidity rates for gallbladder cancer^8^. Cholecystectomy can also be conducted simultaneously with HCC curative resection, because the tumor is adjacent to the gallbladder. Typical clinical manifestations followed by cholecystectomy include dilation of the common bile duct, and increased bile duct pressure and cholestasis, which may cause chronic infections in peripheral liver tissue and even systemic inflammation^9–12^. Chronic inflammation frequently induces necrosis and hepatocyte regeneration, thus resulting in DNA mutations and hepatocyte cancerization^13,14^. Recently, many epidemiological investigations and systematic reviews have addressed the relationship between cholecystectomy and HCC, and most of these studies have indicated that cholecystectomy is associated with a high risk of HCC^15–17^. Moreover, one study by Li et al.^18^ has found that cholecystectomy is associated with a higher risk of early recurrence after curative resection for early-stage HCC. The relationship between cholecystectomy and the prognostic outcomes of patients with HCC after thermal ablation has not been clarified.

Therefore, we conducted a retrospective study with long-term follow-up of consecutive patients with HCC who underwent MWA, by using propensity score matching (PSM) to strengthen causal arguments by reducing potential bias. To our knowledge, this is the first study to consider cholecystectomy as a potential risk factor in analyzing the prognostic outcomes of patients with HCC who underwent thermal ablation.

## Materials and methods

### Patients

This retrospective study was approved by the Institutional Review Board of the Chinese PLA General Hospital and was exempt from requirements to obtain informed consent. Between January 2005 and December 2017, 1,023 consecutive patients with HCC underwent MWA. Of these, 921 patients (754 men and 167 women) were included in this study after meeting the following inclusion criteria: (a) refusal of, or intolerance to, surgical therapy (e.g., cirrhosis or insufficient liver function); (b) a solitary HCC nodule of 5 cm or less, or as many as 3 nodules 3 cm or less, no extrahepatic metastasis or major vessel invasion (Milan criteria); (c) Child-Pugh class of 0 or A; (d) Eastern Cooperative Oncology Group (ECOG) performance status of 0 or 1; and (e) more than 12 months of follow-up time.

The baseline variables were selected according to clinical relevance and the results of previous studies^19,20^, and included age, sex, maximum tumor diameter, number of tumors, cirrhosis type, Child-Pugh class, a-fetoprotein (AFP) concentration, aspartic transaminase (AST) level, alanine aminotransferase (ALT) level, γ-glutamyl transpeptidase (γ-GT) level, albumin (ALB) level, total bilirubin (TBil) level, creatinine (Cre) level, blood platelet (PLT) count, international normalized ratio (INR), neutrophil count, and lymphocyte count. The upper limits of the normal values in our hospital were used as the cut-off values. Preoperative serum tests were performed 1 week before MWA.

### MWA procedure

All MWA procedures were performed by experienced doctors (P.L. and J.Y.) in our department. Color Doppler and grayscale ultrasound were performed to choose the safest access for the antenna. The antenna was connected to the MW generator and inserted into the target area of the tumor. When there were 2 antennas, they were usually deployed in parallel 1–2.5 cm apart. In addition, the antennas were usually deployed parallel to vessels to decrease the damage to large vessels. In general, the microwave energy application was set to 50–60 W for 5–10 min per session. The region of ablation was monitored with real-time ultrasound. MWA emission was stopped when the hyperechoic zone covered the entire tumor, including a safety margin. If necessary, owing to tumor size, multiple overlapping ablations were usually needed to envelope the entire tumor with at least a 5 mm safety margin.

### Follow-up

Contrast-enhanced (CE) imaging (CE-magnetic resonance imaging, CE-computed tomography, or CE ultrasound) was performed within 3 days after the ablation to assess whether complete ablation was achieved. After a residual tumor was recognized, additional ablation was performed to achieve complete coagulation. Patients were followed up at 1 month, every 3 months in the first year after ablation, and then every 6 months in the following years. Patients were also monitored prospectively for recurrence with a standard protocol that included serum AFP and CE imaging. An MR scan of the abdomen was performed every 6 months. Recurrent tumors identified during follow-up were treated with optimal treatment, such as MWA, transcatheter arterial chemoembolization, radiation therapy, targeted therapy, or liver transplantation, according to the recommendations of a multidisciplinary tumor board, on the basis of liver function, patient performance, and recurrent tumor characteristics.

### Definition

HCC diagnosis was confirmed according to the pathologic examination findings or the current practice guidelines of the AASLD^21,22^. Standardized terminology and reporting criteria were used for imaging-guided tumor ablation^23^. Overall survival (OS) was recorded from the local hepatic therapy to the date of death or the date of the last follow-up. Disease-free survival (DFS) was calculated from the beginning of therapy to the time of tumor recurrence or to the time of death or last patient visit. The recurrence of tumors was divided into 3 types: local tumor recurrence, intrahepatic distant recurrence, and extrahepatic recurrence. Local tumor recurrence was defined as the appearance of new tumor foci at the ablative margin after the local eradication of all tumor cells with ablation. Intrahepatic distant recurrence was defined as the detection of the tumor distally from the site of ablation within the liver. Extrahepatic recurrence was defined as the appearance of tumors in extrahepatic organs. Ultrasound-guided fine-needle biopsy was required to confirm the diagnosis when imaging findings were atypical.

### Statistical analysis

All statistical analyses were performed in SPSS Version 22 (College Station, TX, USA). The *t*-test or Mann-Whitney U test was used to assess continuous variables including the maximum tumor diameter, AST level, ALT level, γ-GT level, ALB level, TBil level, Cre level, PLT count, INR, neutrophil count, and lymphocyte count. Fisher’s exact test and χ^2^-test were used to assess the categorical variables including age, sex, number of tumors, AFP level, type of cirrhosis, and Child-Pugh class. Binary logistic regression was performed to achieve 1:2 matching between the cholecystectomy group and the non-cholecystectomy group. A 1:2 nearest-neighbor algorithm was used with a maximum tolerated difference between propensity scores in the range of 0.05. The Kaplan-Meier method was used to evaluate cumulative incidence rates of OS and DFS, and the log-rank test was used to evaluate differences between the cholecystectomy group and the non-cholecystectomy group. Univariable and multivariable Cox proportional hazard models were used to assess the association between possible risk factors for OS and DFS between the non-cholecystectomy group and the cholecystectomy group. The best cut-off value of the γ-GT level was calculated with time-dependent receiver operating characteristic (ROC) curve analysis. Spearman’s correlation analysis was used to assess potential correlations between risk factors. A two-tailed 95% confidence interval (CI) was applied to reveal the accuracy of the hazard ratio (HR), and a *P* value less than 0.05 was considered to indicate a statistically significant difference.

## Results

### Baseline characteristics

**Figure 1:**
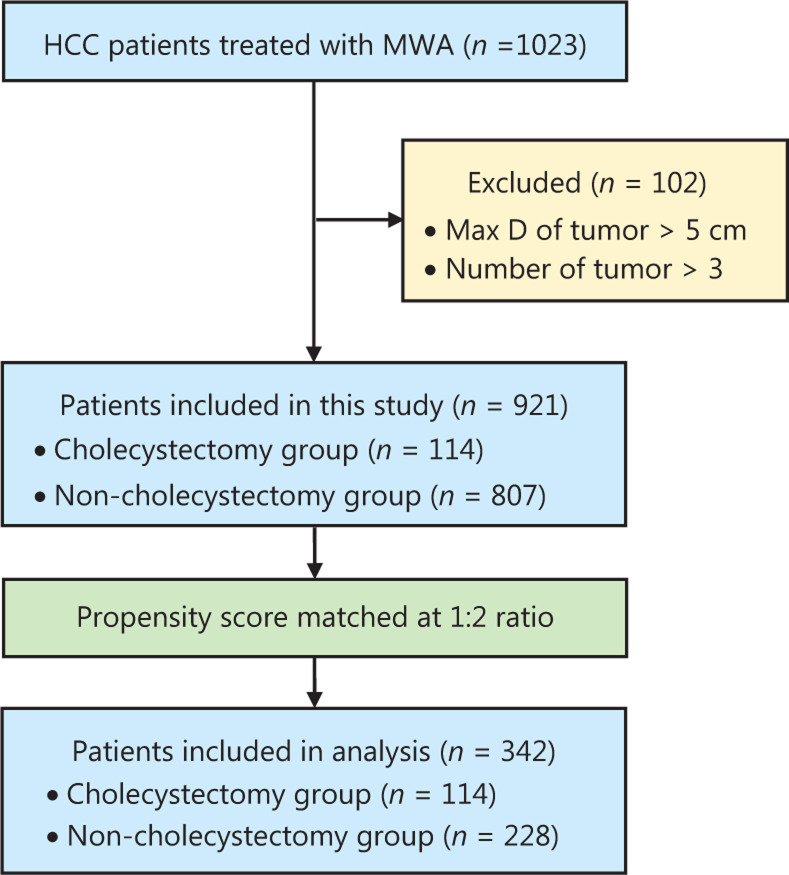
Flow diagram of the patient selection process.

A total of 1,023 patients received MWA for HCC between January 2005 and December 2017 in our department; among them, 102 patients were excluded. A total of 921 patients were included in this study, of whom 114 (12.4%) were in the cholecystectomy group, and 807 (87.2%) were in the non-cholecystectomy group. The reasons for cholecystectomy were as follows: cholelithiasis in 72 patients, gallbladder polyps in 26 patients, acute cholecystitis in 9 patients, and gallbladder adenomyoma in 7 patients. Before PSM, the maximum tumor diameter, number of tumors, AST level, ALT level, and lymphocyte count were significantly different between the 2 groups (**Table 1**). In view of nonrandomized study design and confounding variables, we used PSM for the analysis of observational data to reduce potential bias and performed PSM at a 1:2 ratio. All 114 patients in the cholecystectomy group were successfully matched with 228 patients in the non-cholecystectomy group. No statistically significant differences were observed in the baseline characteristics between the groups after PSM. A flow diagram of the patient selection process is shown in **Figure 1**. The baseline characteristics of patients before and after matching are listed in **Table 1**.

### Comparison of survival

**Figure 2:**
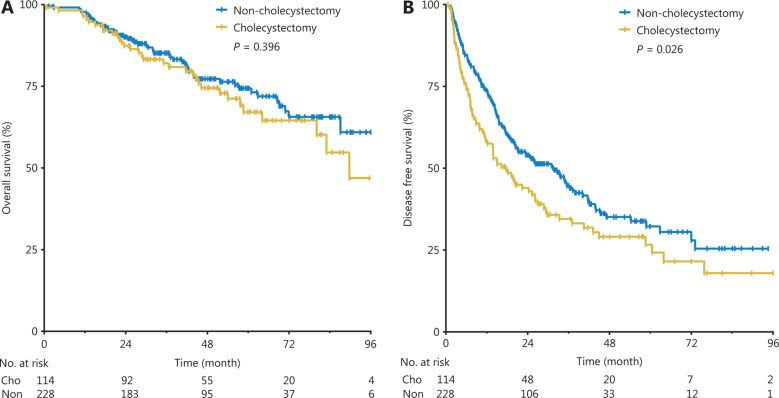
Cumulative OS rates (A) and DFS rates (B) between the non-cholecystectomy group and the cholecystectomy group.

The median follow-up times was 45.8 months (range, 0.6–96.0 months) in the cholecystectomy group and 41.3 months (range, 1.3–96.0 months) in the non-cholecystectomy group (*P* = 0.697). Until the follow-up ended, the death incidence rates were 28.95% and 23.25% for the cholecystectomy group and non-cholecystectomy group, respectively. The cumulative 1-, 3-, and 5-year OS rates were 96.5%, 82.1%, and 67.1% in the cholecystectomy group, and 97.4%, 85.2%, and 74.4% in the non-cholecystectomy group, respectively (*P* = 0.396, **Figure 2A**).

The median DFS time was 16.7 months (range, 0.6–96.0 months) in the cholecystectomy group and 21.4 months (range, 1.3–94.6 months) in the non-cholecystectomy group (*P* = 0.358). Until the follow-up ended, the recurrence rates were 70.2% and 58.8% for the cholecystectomy group and non-cholecystectomy group, respectively. The cumulative 1-, 3-, and 5-year DFS rates were 58.4%, 34.5%, and 26.6% in the cholecystectomy group, and 73.6%, 44.7%, and 32.2% in the non-cholecystectomy group, respectively. The cumulative DFS rates of the non-cholecystectomy group were significantly higher than those of the cholecystectomy group (*P* = 0.026, **Figure 2B**).

### Cox analysis of risk factors associated with OS and DFS

All baseline characteristics of patients with HCC and cholecystectomy were included in univariate and multivariate Cox analyses of potential risk factors associated with survival.

For OS, univariate analysis revealed that age (HR = 1.678, 95% CI 1.089–2.585, *P* = 0.010), number of tumors (2 *vs.* 1: HR = 2.261, 95% CI 1.426–3.587, *P* < 0.001; 3 *vs.* 1: HR = 1.678, 95% CI 1.089–2.585, *P* = 0.003), Child-Pugh class (HR = 2.318, 95% CI 1.158–4.637,* P* = 0.018), and lymphocyte count (HR = 0.074, 95% CI 0.009–0.625,* P* = 0.017) were associated risk factors (**Table 2**). Multivariate analysis revealed that age (HR = 1.778, 95% CI 1.130–2.829, *P* = 0.013) and number of tumors (2 *vs.* 1: HR = 3.010, 95% CI 1.658–5.463, *P* < 0.001; 3 *vs.* 1: HR = 5.981, 95% CI 2.556–13.997, *P* < 0.001) were independent risk factors associated with OS (**Table 2**).

For DFS, univariate analysis revealed that cholecystectomy (HR = 1.367, 95% CI 1.036–1.804, *P* = 0.027), sex (HR = 1.486, 95% CI 1.015–2.176, *P* = 0.041), number of tumors (2 *vs.* 1: HR = 2.332, 95% CI 1.745–3.118, *P* < 0.001; 3 *vs.* 1: HR = 2.522, 95% CI 1.647–3.862, *P* < 0.001), γ-GT level (HR = 1.004, 95% CI 1.002–1.006, *P* < 0.001), and Cre level (HR = 1.002, 95% CI 1.000–1.004, *P* = 0.012) were associated risk factors (**Table 3**). Multivariate analysis revealed that cholecystectomy (HR = 1.364, 95% CI 1.023–1.819, *P* = 0.035), number of tumors (2 *vs.* 1: HR = 2.744, 95% CI 1.925–3.912, *P* < 0.001; 3 *vs.* 1: HR = 3.411, 95% CI 2.021–5.759, *P* < 0.001), and γ-GT level (HR = 1.003, 95% CI 1.000–1.006, *P* = 0.024) were independent risk factors associated with DFS (**Table 3**).

### Subgroup analysis according to the risk factors

**Figure 3:**
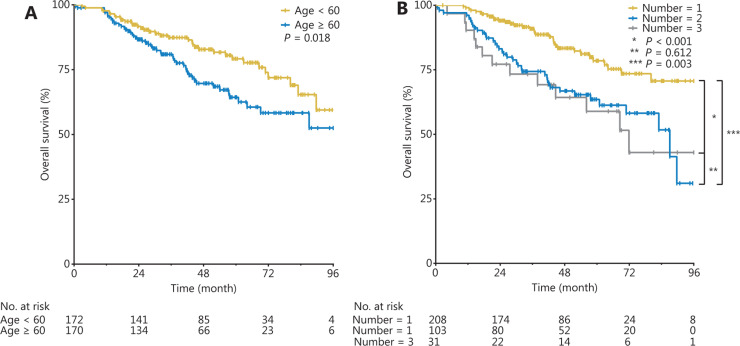
Cumulative OS rates in groups < 60 years and ≥ 60 years of age (A), and in the 1-tumor, 2-tumor, and 3-tumor groups (B).

The cumulative OS rates of the group < 60 years of age were significantly higher than those of the group ≥ 60 years of age (*P* = 0.018, **Figure 3A**). The cumulative OS rates of the 1-tumor group were significantly higher than those of the 2-tumor group (*P* < 0.001, **Figure 3B**) and the 3-tumor group (*P* = 0.003, **Figure 3B**), but there was no statistically significant difference between the 2-tumor group and the 3-tumor group (*P* = 0.612, **Figure 3B**).

**Figure 4:**
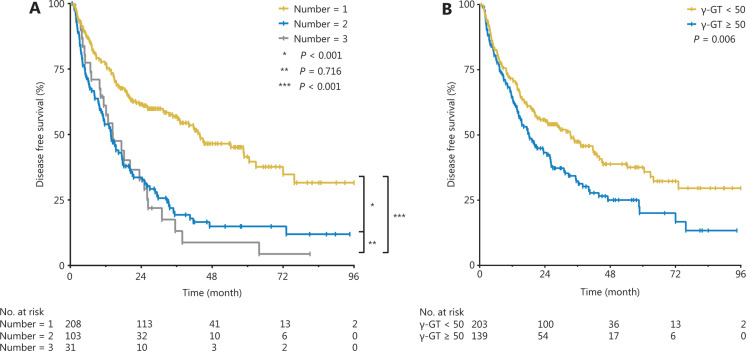
Cumulative DFS rates in the γ-GT < 50 U/L and γ-GT ≥ 50 U/L groups (A) and in the 1-tumor, 2-tumor, and 3-tumor groups (B).

Furthermore, the cumulative DFS rates of the 1-tumor group were significantly higher than those of the 2-tumor group (*P* < 0.001, **Figure 4A**) and 3-tumor group (*P* < 0.001, **Figure 4A**), but there was no statistically significant difference between the 2-tumor group and the 3-tumor group (*P* = 0.716, **Figure 4A**). The cumulative DFS rates of the γ-GT < 50 U/L group were significantly higher than those of the γ-GT ≥ 50 U/L group (*P* = 0.006, **Figure 4B**).

**Figure 5:**
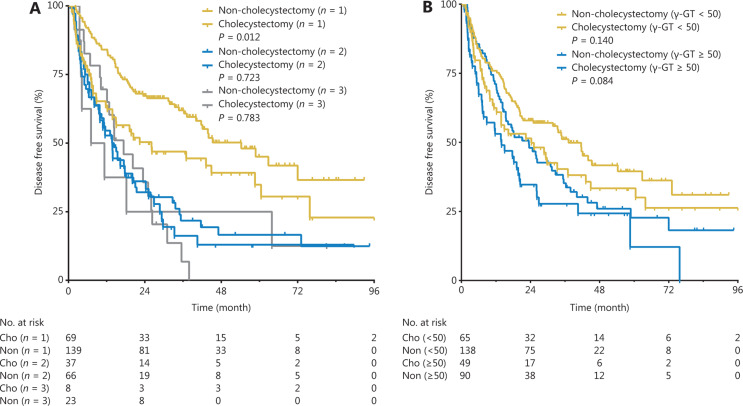
Subgroup analysis according to the number of tumors (A) and γ-GT levels (B) in the non-cholecystectomy group and the cholecystectomy group.

In the subgroup of patients with 1 tumor, the 1-, 3-, and 5-year DFS rates were 63.8%, 46.9%, and 34.8% in the cholecystectomy group, and 84.1%, 61.9%, and 45.0% in the non-cholecystectomy group, respectively (*P* = 0.012, **Figure 5A**). In the subgroup of patients with 2 or 3 tumors, no statistically significant differences in DFS rates were observed between the groups (*P* = 0.723 and *P* = 0.783, respectively, **Figure 5A**).

When the cut-off value of γ-GT was 50 U/L (the upper limit of normal values in our hospital), subgroup analysis revealed no statistically significant differences in DFS rates between the cholecystectomy group and the non-cholecystectomy group, not only for patients with γ-GT < 50 U/L (*P* = 0.140, **Figure 5B**) but also for patients with γ-GT ≥ 50 U/L (*P* = 0.084, **Figure 5B**).

**Figure 6:**
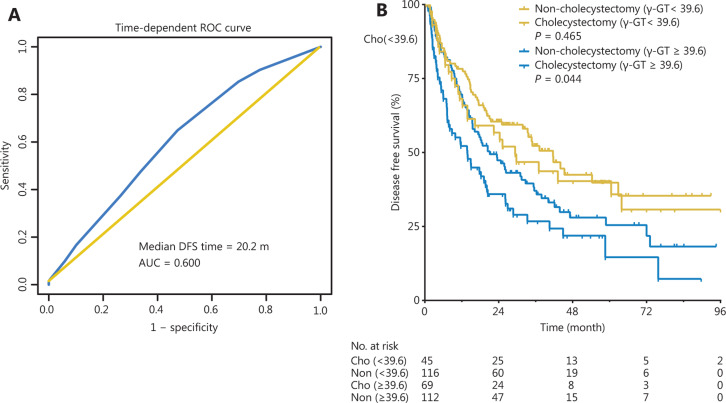
Time-dependent ROC curve for the cut-off value of the γ-GT level (A); subgroup analysis according to the γ-GT level (B) of the non-cholecystectomy and cholecystectomy groups.

Given that the γ-GT level was an independent risk factor associated with DFS, time-dependent ROC curve analysis indicated that 39.6 U/L was the best cut-off value of the γ-GT level for predicting median DFS at 20.2 months (area under the curve = 0.600, *P* < 0.05) (**Figure 6A**). Then 342 patients were divided into 2 groups: a low γ-GT group (< 39.6 U/L, *n* = 161, 47.1%) and a high γ-GT group (≥ 39.6 U/L, *n* = 181, 52.9%). The DFS rates of the low γ-GT group were significantly higher than those of the high γ-GT group (*P* < 0.001, **Figure 6B**). In the subgroup of patients with γ-GT ≥ 39.6 U/L, the DFS rates of the non-cholecystectomy group were significantly higher than those of the cholecystectomy group (*P* = 0.044, **Figure 6B**); however, in the subgroup of patients with γ-GT < 39.6 U/L, there were no statistically significant differences in the DFS rates between the groups (*P* = 0.465, **Figure 6B**).

### Comparison of recurrence

**Figure 7:**
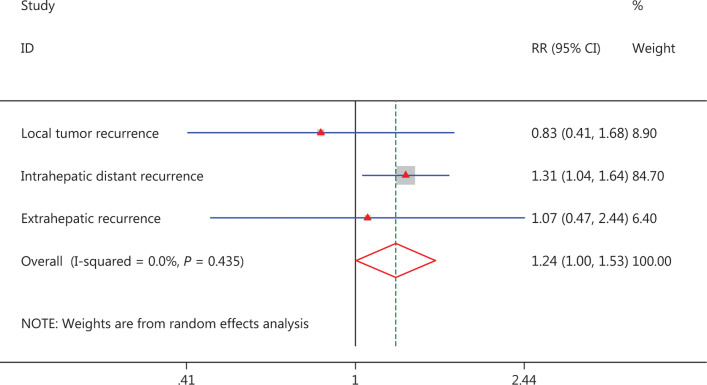
Forest plot for the comparison of recurrence.

As mentioned above, the total recurrence rates were 70.2% (80/114) in the cholecystectomy group and 58.8% (134/228) in the non-cholecystectomy group, and a higher recurrence rate was observed in the cholecystectomy group (*P* = 0.040); the local tumor recurrence rates were 8.8% (10/114) and 10.5% (24/228), respectively, and there was no statistically significant difference between the groups (*P* = 0.609). The intrahepatic distant recurrence rates were 54.4% (62/114) and 41.7% (95/228) in the cholecystectomy group and non-cholecystectomy group, respectively, and higher recurrence rates were observed in the cholecystectomy group (*P* = 0.026). The extrahepatic recurrence rates were 7.0% (8/80) and 4.4% (15/134) in the cholecystectomy group and non-cholecystectomy group, respectively, and no statistically significant difference was observed between the groups (*P* = 0.879) (**Figure 7**).

### Analysis of correlation between cholecystectomy and ***γ***-GT level

**Figure 8:**
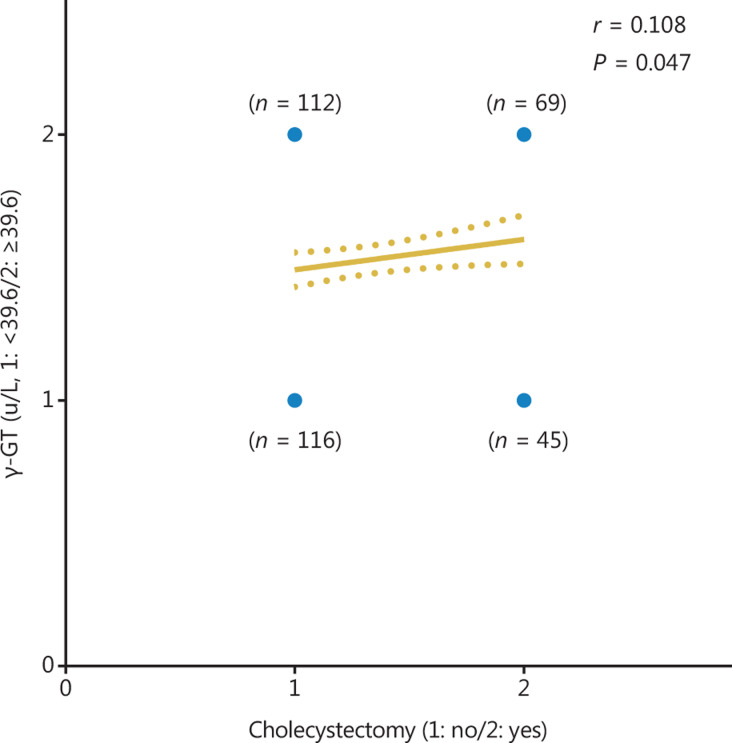
Correlation between cholecystectomy and γ-GT level.

As mentioned above, both cholecystectomy and γ-GT levels were independent risk factors for DFS and were associated with bile metabolism and liver inflammation. Therefore, we performed Spearman’s correlation analysis and found a significantly positive correlation between cholecystectomy and γ-GT levels (*r* = 0.108, 95% CI −0.001–0.214, *P* = 0.047, **Figure 8**).

### Analysis of time from cholecystectomy to recurrence

**Figure 9:**
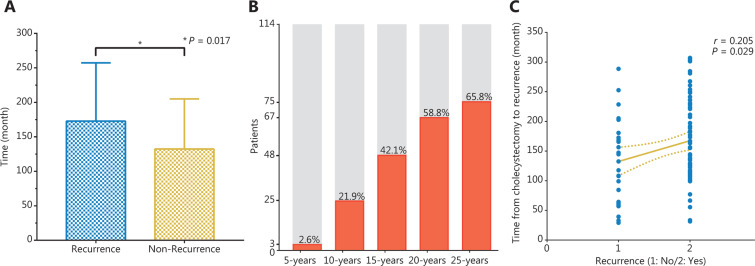
T test for time from cholecystectomy to recurrence in recurrence group and time from cholecystectomy to death or the ending of follow-up in non-recurrence group (A); proportion of patient recurrence from the time of cholecystectomy (B); correlation between recurrence and the time from cholecystectomy to recurrence (C).

In the cholecystectomy group (*n* = 114), patients were divided into a recurrence subgroup (*n* = 80) and non-recurrence subgroup (*n* = 34). The time from cholecystectomy to recurrence was 172.8 ± 84.5 months (median 144.6 months; range 31.5–307.0 months) in the recurrence subgroup, and the mean time from cholecystectomy to death or the ending of follow-up was 132.5 ± 72.6 months (median 138.6 months; range 29.2–288.6 months) in the non-recurrence subgroup; a statistically significant difference was observed between the groups (*P* = 0.017, **Figure 9A**). The 5-, 10-, 15-, 20-, and 25-year recurrence rates from the time of cholecystectomy were 2.6%, 21.9%, 42.1%, 58.8%, and 65.8%, respectively (**Figure 9B**). By Spearman’s correlation analysis, we found a significantly positive correlation between recurrence and the time from cholecystectomy to recurrence (*r* = 0.205, 95% CI 0.016–0.379, *P* = 0.029, **Figure 9C**).

### Complications

The complication rates associated with MWA were 3.3% (30/921) in the overall data (11 in the cholecystectomy group; 19 in the non-cholecystectomy group, *P* = 0.685). Complications that may be associated with cholecystectomy (such as liver abscess and bile leakage) were not significantly more frequent in the cholecystectomy group than in the non-cholecystectomy group (1.8% in the cholecystectomy group *vs.* 1.3% in the non-cholecystectomy group, *P* = 0.750).

## Discussion

HCC is an inflammation-related cancer in which more than 90% of cases have a background of hepatic injury or chronic inflammation, such as hepatitis B or C virus infection, cirrhosis, cholestasis, and cholecystitis^24–26^. Cholecystectomy-induced cholestasis is the major cause of the intrahepatic chronic inflammatory environment, which is considered to increase the risk and recurrence of HCC^24^. However, whether cholecystectomy influences the prognostic outcomes of patients with HCC undergoing thermal ablation has not yet been clarified.

Cholecystectomy was found to be an independent risk factor associated with DFS in this study (*P* = 0.035), and the recurrence rates in the cholecystectomy group were significantly higher than those in the non-cholecystectomy group (*P* = 0.034). High recurrence rate is considered the primary reason for the dismal oncological outcomes in HCC^27^. Some studies^28–30^ have demonstrated that removal of the gallbladder is always accompanied by dysfunction in the sphincter of Oddi, thus potentially leading to dilation of the common bile duct, elevated bile duct pressure, subsequent cholestasis, and chronic inflammation. The intrahepatic chronic inflammatory microenvironment is the most critical factor associated with HCC, and inflammation-induced neoangiogenesis, inflammation-related cytokines, chemokines, and growth factors all play critical roles in cancer recurrence and metastasis. Moreover, the mean time from cholecystectomy to recurrence was 172.8 ± 84.5 months, and a statistically significant difference was observed in this group compared with time from cholecystectomy to death or the ending of follow-up in non-recurrence group (*P* = 0.017). The 5-, 10-, 15-, 20-, and 25-year recurrence rates from the time of cholecystectomy were 2.6%, 21.9%, 42.1%, 58.8%, and 65.8%, respectively, and significantly positive correlations were observed between recurrence and the time from cholecystectomy to recurrence (*r* = 0.205, 95% CI 0.016–0.379, *P* = 0.029), thus indicating an increasing trend of recurrence over time. Lagergren et al.^16^ have reported a significantly greater overall risk of HCC in patients who have undergone cholecystectomy than in the corresponding background population (standardized incidence ratio = 1.24, 95% CI 1.11–1.38), and the risk gradually increases with longer follow-up time (*P* for trend = 0.003). Our findings revealed that the chronic inflammation introduced by cholecystectomy plays an important role in the carcinogenic process.

In addition to cholecystectomy, the γ-GT level was found to be an independent risk factor associated with DFS (*P* = 0.024). Elevated γ-GT levels result from both alcoholic and non-alcoholic fatty liver disease, cholestatic liver disease, and induction by drugs such as phenytoin. γ-GT has been widely applied for detection and diagnosis of liver disease, which is a nearly ubiquitous epithelial enzyme that is responsible for the catabolism of extracellular glutathione. In addition to its association with liver disease, γ-GT has been associated with high all-cause mortality, cardiovascular disease incidence and death, diabetes, and cancer incidence and death^31–33^. Some studies^34–36^ have reported that γ-GT levels have significant prognostic value as a risk factor for HCC, in agreement with our findings. The synthesis of γ-GT is excessive in patients with HCC; moreover, cholecystectomy-related cholestasis may block the excretion of γ-GT, and the blocked γ-GT then returns to the blood and is accompanied by bile, thus increasing serum γ-GT levels. Our findings revealed a positive correlation between cholecystectomy and γ-GT levels (*r* = 0.108, 95% CI −0.001–0.214, *P* = 0.047). The above results suggested that patients with HCC who underwent cholecystectomy and had high γ-GT levels were prone to recurrence after thermal ablation.

In this study, the median follow-up time in the cholecystectomy group and non-cholecystectomy group was 45.8 and 41.3 months, respectively (*P* = 0.697). However, no statistically significant difference in OS rates was observed between the cholecystectomy group and the non-cholecystectomy group (*P* = 0.396). The reason for this finding may be that most patients with recurrence probably benefitted from our multimodal therapy and timely comprehensive management. A phase III randomized controlled trial examining MWA for patients with HCC^37^ has reported 1-, 3-, and 5-year OS rates of 96.4%, 81.9%, and 67.3%, respectively, findings similar to our oncological outcomes.

Several limitations in our study must be noted. First, our retrospective study design may potentially have introduced selection bias. Although we attempted to simulate randomization by using PSM analysis, there remains a possibility of uncontrolled confounding factors. Second, because this was a single-center study, and there was no universal consensus of MWA, our results might not be consistently reproducible in other settings. Despite these limitations, to our knowledge, no previous study has addressed a possible relationship between cholecystectomy and increased risk of recurrence in patients with HCC after thermal ablation. Our findings may help validate the association between cholecystectomy and the prognostic outcomes of patients with HCC who underwent thermal ablation.

In summary, cholecystectomy is an independent prognostic factor associated with the DFS of patients with HCC who underwent thermal ablation. Though it is too early to consider any potential clinical recommendations, we will continue to focus on patients with HCC who underwent cholecystectomy and perform further medical research to explore the relevant mechanisms.

## Figures and Tables

**Table 1:** Baseline characteristics of patients before and after matching

Characteristics		All patients (*n* = 921)			Patients after matching at 1:2 ratio (*n* = 342)		
		Cholecystectomy	Non-cholecystectomy	* P*-value	Cholecystectomy	Non-cholecystectomy	*P*
*n* (%)		114 (12.38%)	807 (87.62%)		114 (33.33%)	392 (66.67%)	
Age (years)	Mean ± SD	59.21 ± 10.13	58.35 ± 10.74	0.422	59.21 ± 10.13	59.21 ± 10.88	0.997
	(Range)	(35.00–87.00)	(24.00–91.00)		(35.00–87.00)	(35.00–88.00)	
	<60	58 (50.88%)	436 (54.03%)	0.528	58 (50.88%)	114 (50.00%)	0.878
	≥60	56 (49.12%)	371 (45.97%)		56 (49.12%)	114 (50.00%)	
Sex	Male	93 (81.58%)	661 (81.91%)	0.932	93 (81.58%)	187 (82.02%)	0.921
	Female	21 (18.42%)	146 (18.09%)		21 (18.42%)	41 (17.98%)	
Max D (cm)	Mean ± SD	2.46 ± 1.00	2.74 ± 1.06	0.009*	2.46 ± 1.00	2.35 ± 0.95	0.348
	(Range)	(0.90–5.00)	(0.80–5.00)		(0.90–5.00)	(0.80–5.00)	
Number	1	69 (60.53%)	586 (72.61%)	0.027*	69 (60.53%)	180 (60.96%)	0.576
	2	37 (32.46%)	177 (21.93%)		37 (32.46%)	125 (28.95%)	
	3	8 (7.02%)	44 (5.45%)		8 (7.02%)	87 (10.09%)	
Cirrhosis	HBV	88 (77.20%)	661 (81.91%)	0.224	88 (77.20%)	191 (83.77%)	0.287
	HCV	13 (11.40%)	83 (10.29%)		13 (11.40%)	16 (7.02%)	
	AH	0 (0.00%)	8 (0.99%)		0 (0.00%)	0 (0.00%)	
	No	13 (11.40%)	55 (6.82%)		13 (11.40%)	21 (9.21%)	
Child-Pugh class	A	109 (95.61%)	779 (96.53%)	0.622	109 (95.61%)	215 (94.30%)	0.607
	B	5 (4.39%)	28 (3.47%)		5 (4.39%)	13 (5.70%)	
AFP (ng/mL)	<20	70 (61.40%)	467 (57.87%)	0.474	70 (61.40%)	138 (60.53%)	0.876
	≥20	44 (38.60%)	340 (42.13%)		44 (38.60%)	90 (39.47%)	
AST (U/L)	Mean ± SD	28.33 ± 15.69	33.89 ± 25.20	0.022*	28.33 ± 15.69	29.34 ± 16.80	0.596
	(Range)	(12.20–99.00)	(10.70–408.40)		(12.20–99.00)	(12.30–110.60)	
ALT (U/L)	Mean ± SD	28.53 ± 18.16	33.59 ± 25.23	0.039*	28.53 ± 18.16	29.43 ± 19.16	0.674
	(Range)	(4.00–108.00)	(4.30–229.10)		(4.00–108.00)	(7.70–127.20)	
γ-GT (U/L)	Mean ± SD	62.33 ± 50.96	78.34 ± 117.01	0.150	62.33 ± 50.96	58.45 ± 53.48	0.521
	(Range)	(11.30–337.80)	(10.00–1789.20)		(11.30–337.80)	(10.00–352.30)	
ALB (g/L)	Mean ± SD	39.99 ± 4.46	39.35 ± 4.67	0.169	39.99 ± 4.46	39.85 ± 4.76	0.800
	(Range)	(26.00–54.10)	(19.80–68.00)		(26.00–54.10)	(23.40–68.00)	
TBIL (μmol/L)	Mean ± SD	15.42 ± 8.24	16.81 ± 9.40	0.134	15.42 ± 8.24	15.63 ± 8.65	0.827
	(Range)	(4.50–43.10)	(1.40–79.60)		(4.50–43.10)	(1.40–70.00)	
CRE (μmol/L)	Mean ± SD	74.78 ± 27.62	73.88 ± 47.26	0.844	74.78 ± 27.62	77.38 ± 70.85	0.705
	(Range)	(4.11–281.00)	(4.40–1034.70)		(4.11–281.00)	(4.70–1034.70)	
PLT (10^9^/L)	Mean ± SD	120.90 ± 50.25	120.67 ± 61.15	0.969	120.90 ± 50.25	121.64 ± 60.71	0.911
	(Range)	(26.00–309.00)	(20.00–459.00)		(26.00–309.00)	(20.00–336.00)	
INR (10^9^/L)	Mean ± SD	1.65 ± 5.89	1.32 ± 4.14	0.458	1.65 ± 5.89	1.14 ± 0.15	0.195
	(Range)	(0.82–64.00)	(0.87–117.00)		(0.82–64.00)	(0.90–1.82)	
NEUT (10^9^/L)	Mean ± SD	0.57 ± 0.10	0.55 ± 0.10	0.113	0.57 ± 0.10	0.57 ± 0.11	0.746
	(Range)	(0.40–0.91)	(0.06–0.94)		(0.40–0.91)	(0.06–0.94)	
LYMPH (10^9^/L)	Mean ± SD	0.32 ± 0.10	0.33 ± 0.09	0.010*	0.32 ± 0.10	0.32 ± 0.10	0.698
	(Range)	(0.05–0.76)	(0.05–0.65)		(0.05–0.76)	(0.05–0.60)	

*P < 0.05.

**Table 2:** Cox analyses of risk factors associated with OS

Variables (OS)		Univariate analysis			Multivariate analysis		
		HR	95% CI	*P*	HR	95% CI	*P*
Cholecystectomy	No	1.207	0.781–1.864	0.397	1.160	0.730–1.843	0.530
	Yes						
Age (years)	<60	1.678	1.089–2.585	0.010*	1.788	1.130–2.829	0.013*
	≥60						
Sex	Female	1.350	0.733–2.486	0.335	1.575	0.808–3.073	0.182
	Male						
Max D (cm)		1.011	0.807–1.268	0.922	1.419	0.962–1.967	0.519
Number	1	1.000	–	–	1.000	–	–
	2	2.261	1.426–3.587	<0.001*	3.010	1.658–5.463	<0.001*
	3	2.590	1.369–4.900	0.003*	5.981	2.556–13.997	<0.001*
Cirrhosis	No	1.000	–	–	1.000	–	–
	HBV	0.552	0.283–1.076	0.081	0.558	0.275–1.132	0.106
	HCV	0.740	0.300–1.824	0.513	0.637	0.228–1.775	0.388
	AH	–	–	–	–	–	–
Child-Pugh class	A	2.318	1.158–4.637	0.018*	2.001	0.907–4.414	0.086
	B						
AFP (ng/mL)	<20	1.128	0.736–1.729	0.581	0.990	0.613–1.599	0.967
	≥20						
AST (U/L)		1.004	0.993–1.015	0.487	1.008	0.987–1.030	0.450
ALT (U/L)		0.999	0.988–1.011	0.891	0.992	0.974–1.010	0.399
γ-GT (U/L)		1.003	0.999–1.006	0.137	1.000	0.996–1.005	0.936
ALB		1.001	0.959–1.044	0.971	1.019	0.968–1.072	0.478
TBIL		0.994	0.970–1.019	0.636	0.985	0.956–1.014	0.307
CRE		0.999	0.995–1.004	0.810	0.998	0.991–1.004	0.482
PLT		0.999	0.995–1.003	0.490	0.999	0.994–1.004	0.611
INR		1.009	0.977–1.041	0.585	1.007	0.972–1.042	0.716
NEUT		6.520	1.077–39.481	0.041	0.881	0.009–86.150	0.957
LYMPH		0.074	0.009–0.625	0.017*	0.037	0.000–6.343	0.209

*P < 0.05.

**Table 3:** Cox analyses of risk factors associated with DFS

Variables (DFS)		Univariate analysis			Multivariate analysis		
		HR	95% CI	*P*	HR	95% CI	*P*
Cholecystectomy	No	1.367	1.036–1.804	0.027*	1.364	1.023–1.819	0.035*
	Yes						
Age (years)	<60	1.306	0.998–1.711	0.052	1.237	0.929–1.646	0.146
	≥60						
Sex	Female	1.486	1.015–2.176	0.041*	1.365	0.910–2.046	0.132
	Male						
Max D (cm)		0.962	0.835–1.109	0.595	1.261	0.859–1.503	0.509
Number	1	1.000	–	–	1.000	–	–
	2	2.332	1.745–3.118	<0.001*	2.744	1.925–3.912	<0.001*
	3	2.522	1.647–3.862	<0.001*	3.411	2.021–5.759	<0.001*
Cirrhosis	No	1.000	–	–	1.000	–	–
	HBV	1.337	0.789–2.266	0.286	1.313	0.755–2.285	0.335
	HCV	1.825	0.940–3.543	0.075	1.511	0.730–3.126	0.266
	AH	–	–	–	–	–	–
Child-Pugh class	A	1.145	0.639–2.051	0.649	1.000	0.514–1.945	0.999
	B						
AFP (ng/mL)	<20	1.203	0.916–1.579	0.183	1.147	0.854–1.540	0.362
	≥20						
AST (U/L)		1.000	0.992–1.008	0.991	1.001	0.987–1.014	0.930
ALT (U/L)		1.000	0.993–1.007	0.893	0.992	0.981–1.004	0.183
γ-GT (U/L)		1.004	1.002–1.006	<0.001*	1.003	1.000–1.006	0.024*
ALB		1.026	0.997–1.055	0.183	1.016	0.979–1.054	0.397
TBIL		0.989	0.973–1.006	0.205	0.991	0.972–1.011	0.384
CRE		1.002	1.000–1.004	0.012*	1.001	1.000–1.003	0.118
PLT		0.999	0.996–1.001	0.247	0.998	0.995–1.001	0.277
INR		0.485	0.191–1.231	0.128	0.684	0.164–2.856	0.602
NEUT		1.519	0.421–5.4884	0.524	1.287	0.066–25.185	0.868
LYMPH		0.555	0.135–2.280	0.414	0.431	0.020–9.203	0.590

*P < 0.05.
